# African Mountain Thistles: Three New Genera in the *Carduus-Cirsium* Group

**DOI:** 10.3390/plants12173083

**Published:** 2023-08-28

**Authors:** Lucía D. Moreyra, Núria Garcia-Jacas, Cristina Roquet, Jennifer R. Ackerfield, Turan Arabacı, Carme Blanco-Gavaldà, Christian Brochmann, Juan Antonio Calleja, Tuncay Dirmenci, Kazumi Fujikawa, Mercè Galbany-Casals, Tiangang Gao, Abel Gizaw, Javier López-Alvarado, Iraj Mehregan, Roser Vilatersana, Bayram Yıldız, Frederik Leliaert, Alexey P. Seregin, Alfonso Susanna

**Affiliations:** 1 Botanic Institute of Barcelona (IBB), CSIC-Ajuntament de Barcelona, Pg. Migdia, s.n., 08038 Barcelona, Spain; luciad.moreyra@ibb.csic.es (L.D.M.); ngarciajacas@ibb.csic.es (N.G.-J.); vilatersana@ibb.csic.es (R.V.); 2 Systematics and Evolution of Vascular Plants (UAB)—Associated Unit to CSIC by IBB, Autonomous University of Barcelona, 08193 Cerdanyola del Vallès, Spain; cristina.roquet@uab.cat (C.R.); carme.blanco@uab.cat (C.B.-G.); merce.galbany@uab.cat (M.G.-C.);; 3 Department of Biology, Colorado State University, Fort Collins, CO 80523, USA; jennifer.ackerfield@botanicgardens.org; 4 Department of Pharmaceutical Botany, Faculty of Pharmacy, Inönü University, 44280 Malatya, Türkiye; turan.arabaci@inonu.edu.tr; 5 Natural History Museum, University of Oslo, Blindern, 0318 Oslo, Norway; christian.brochmann@nhm.uio.no (C.B.); aabegiz3@gmail.com (A.G.); 6 Department of Biology, Autonomous University of Madrid, 28049 Madrid, Spain; juan.calleja@uam.es; 7 Centro de Investigación en Biodiversidad y Cambio Global, Universidad Autónoma de Madrid, 28049 Madrid, Spain; 8 Department of Biology, Faculty of Necatibey Education, Balıkesir University, 10145 Balıkesir, Türkiye; dirmenci@balikesir.edu.tr; 9 Kochi Prefectural Makino Botanical Garden, 4200-6, Godaisan, Kochi 781-8125, Japan; saussure@makino.or.jp; 10 State Key Laboratory of Systematic and Evolutionary Botany, Institute of Botany, Chinese Academy of Sciences, Beijing 100093, China; gaotg@ibcas.ac.cn; 11 Department of Plant Biology and Biodiversity Management, Addis Ababa University, Addis Ababa P.O. Box 3434, Ethiopia; 12 Department of Biology, Science and Research Branch, Islamic Azad University, Tehran 1477893855, Iran; iraj@daad-alumni.de; 13 Ismail Cem Street, No. 35, Yenikale District, 35320 Narlidere Türkiye; bayramyildiz36@hotmail.com; 14 Meise Botanic Garden, Nieuwelaan 38, 1860 Meise, Belgium; frederik.leliaert@plantentuinmeise.be; 15 Faculty of Biology, M. V. Lomonosov Moscow State University, 119991 Moscow, Russia; botanik.seregin@gmail.com

**Keywords:** Afroalpine, Afromontane, Carduinae, *Carduus-Cirsium* group, Hyb-Seq, systematics

## Abstract

The floras on the highest mountains in tropical eastern Africa are among the most unique floras in the world. Despite the exceptionally high concentration of endemic species, these floras remain understudied from an evolutionary point of view. In this study, we focus on the *Carduus-Cirsium* group (subtribe Carduinae) to unravel the evolutionary relationships of the species endemic to the tropical Afromontane and Afroalpine floras, aiming to improve the systematics of the group. We applied the Hyb-Seq approach using the Compositae1061 probe set on 190 samples (159 species), encompassing representatives of all genera of Carduinae. We used two recently developed pipelines that enabled the processing of raw sequence reads, identification of paralogous sequences and segregation into orthologous alignments. After the implementation of a missing data filter, we retained sequences from 986 nuclear loci and 177 plastid regions. Phylogenomic analyses were conducted using both concatenated and summary-coalescence methods. The resulting phylogenies were highly resolved and revealed three distinct evolutionary lineages consisting of the African species traditionally referred to as *Carduus* and *Cirsium*. Consequently, we propose the three new genera *Afrocarduus*, *Afrocirsium* and *Nuriaea*; the latter did notably not belong to the *Carduus*-*Cirsium* group. We detected some incongruences between the phylogenies based on concatenation vs. coalescence and on nuclear vs. plastid datasets, likely attributable to incomplete lineage sorting and/or hybridization.

## 1. Introduction

The current biodiversity crisis entails numerous consequences and unprecedented challenges [1,2], emphasizing the urgent need to enhance our systematic understanding of biodiversity, as it serves as a crucial tool for its conservation [3]. Despite hosting multiple biodiversity hotspots, the least studied areas of the world, from an evolutionary perspective, are located in the tropics [4,5]. It is particularly urgent to fill such a knowledge gap given the disproportionately high extinction rates experienced by tropical regions [6] and the significant anthropogenic pressures they face, such as extensive land-use changes [7]. 

The Afromontane and Afroalpine archipelagos host unique tropical floras, which are part of the Afrotemperate biome [8,9]. These tropical Afrotemperate flora thrive in humid and temperate isolated refuges on the highest African mountains, which are located primarily in the eastern part of the continent [10]. Both the Afromontane flora (below the treeline) and the Afroalpine flora (above the treeline) harbor exceptionally high concentrations of endemic species (c. 75%, [11,12]). Most of the research on plant evolution has focused on the Afroalpine flora (reviewed in Brochmann et al., 2021 [13]), leaving the Afromontane flora understudied. To address this knowledge gap, our study aims to investigate the *Carduus-Cirsium* group (subtribe Carduinae), which includes species that occur both below and above the treeline and are mostly endemic to tropical eastern Africa. 

The *Carduus-Cirsium* group as currently defined (e.g., Susanna and Garcia-Jacas, 2007 [14]) encompasses ca. 550 species in six genera (Table 1): *Carduus* L. (ca. 100 species, Eurasia and eastern Africa), *Cirsium* Mill. (ca. 450 species, Eurasia, North America, northern and eastern Africa), *Notobasis* (Cass.) Cass. (one species, Eurasia), *Picnomon* Adans. (one species, Eurasia), *Silybum* Adans. (two species, subcosmopolitan) and *Tyrimnus* Cass. (one species, Mediterranean). The *Carduus-Cirsium* group remains the most taxonomically problematic group in the Carduinae [15,16]. Current species of *Cirsium* have been classified into at least 16 different genera, and its delimitation is still controversial, leading to multiple reclassifications of many Carduinae into different genera [15,17,18,19,20]. These delimitation difficulties are confounded by (1) low phylogenetic resolution in previous studies based on nuclear ribosomal transcribed spacers (ETS and ITS), a few low-copy nuclear genes and plastid regions [17,19,20,21,22,23,24,25]; (2) morphological convergence in response to similar environmental conditions [26,27]; and (3) hybridization as a recurrent mechanism of speciation within the tribe Cardueae [28]. 

Despite recent research efforts [19,20], *Cirsium* and *Carduus* remain the most problematic genera. The recent taxonomic proposal for the *Carduus-Cirsium* group (20) split *Cirsium* into four genera, accepting Cassini’s subgenus *Lophiolepis* at the generic level, reinstating the genus *Epitrachys* (DC. Ex Duby) K.Koch and describing the hybrid genus x*Lophiocirsium* Del Guacchio, Bureš, Iamonico & P. Caputo, mainly based on the weakly supported molecular results from Ackerfield et al. (2020) [19]. However, both studies lacked sufficient sampling across the *Carduus-Cirsium* group. For example, Afromontane *Cirsium* species were not included despite their distinct morphological differences from all other species in *Cirsium*.

*Carduus*, the second most speciose genus, is monophyletic only when excluding subgenus *Afrocarduus* Kazmi [29] from the phylogenetic analyses [17,19,25]. Subgenus *Afrocarduus* is endemic to the mountains of tropical eastern Africa and is a good example of the adaptation, radiation and evolution of montane and alpine species. It includes both widespread taxa growing across several mountain regions and narrow endemics restricted to one massif, all of them between 1600 and 4600 m.a.s.l. [29,30,31]. *Afrocarduus* species exhibit a combination of morphological and karyological characters typical of both *Carduus* and *Cirsium*. Species delimitation within this group is highly controversial. The two main taxonomic treatments differ conspicuously in the number of accepted species: 22 in Kazmi (1963) [29] and only 9 in Jeffrey (1968) [31]. 

The controversies surrounding the delineation of *Cirsium* as well as the unresolved relationships of the Afromontane species underscore the need for a new approach to resolve the phylogeny and systematics of the *Carduus-Cirsium* group. Previous studies suffered from limited taxon and gene sampling, resulting in poorly resolved phylogenies. Next-Generation Sequencing (NGS) methods offer improved resolution in phylogenetic reconstructions at all taxonomic levels [32,33,34,35]. Here, we compiled a comprehensive sampling of all the genera in the subtribe and applied a Hyb-Seq approach using a probe set targeting exons of 1061 orthologous loci developed for Compositae [36], which also enabled the retrieval of complete plastid genome sequences. The three main objectives of our study are to (1) infer the first phylogeny of the subtribe Carduinae that includes a comprehensive taxonomical and biogeographical sampling; (2) establish a delimitation of the genera with a particular focus on the *Carduus-Cirsium* group; and (3) unravel the evolutionary origins of the Afromontane and Afroalpine species.

## 2. Results

### 2.1. Target Loci Recovery

Out of 1064 loci of the target reference, 918 were recovered with a mean coverage of the retained contigs of 45.38. The customized reference after splitting the paralogous sequences into orthologous alignments comprised a total of 1407 loci, of which 507 alignments were generated after splitting the paralogous ones. After mapping and filtering by missing data and sample presence, 986 loci were retained, of which 775 originally contained only orthologous sequences, whereas 211 originated from the splitting of paralogous sequences into orthologous alignments. Sequence divergence values between 8.6 and 22.7% were considered to indicate paralogy (Figure S1). The average alignment length was 250 bp (88–719), the average parsimony site length was 53 bp (6–191), the average variable site length was 76 bp (12–275) and the average missing data was 3.69% (0–65%). 

For the concatenated analysis, the nuclear and plastid supermatrix had a length of 247,251 bp and 117,823 bp, respectively. The coalescence analysis was based on 986 alignments, and a gene tree was calculated for each of them. For the plastid data, we retrieved a total of 183 regions, of which we retained 177 after filtering for missing data and sample presence: the final plastid dataset included 30 tRNAs, 4 rRNAs, 79 coding genes (including 13 introns) and 64 intergenic regions.

### 2.2. Phylogenetic Relationships

#### 2.2.1. Nuclear Dataset

**Figure 1:**
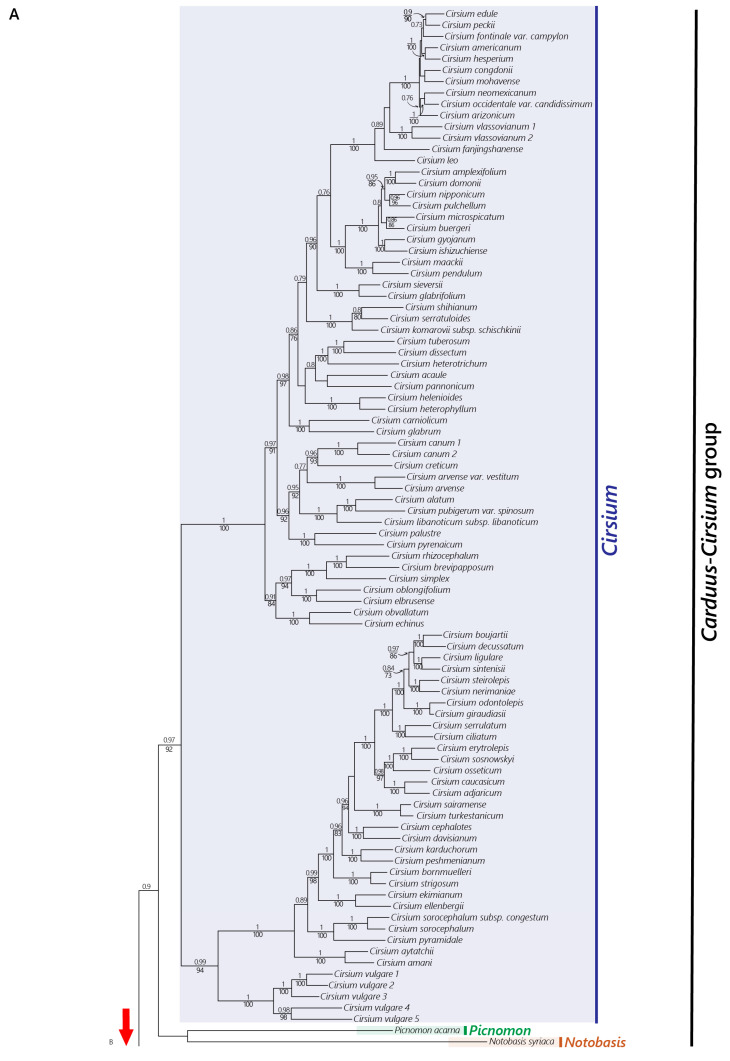

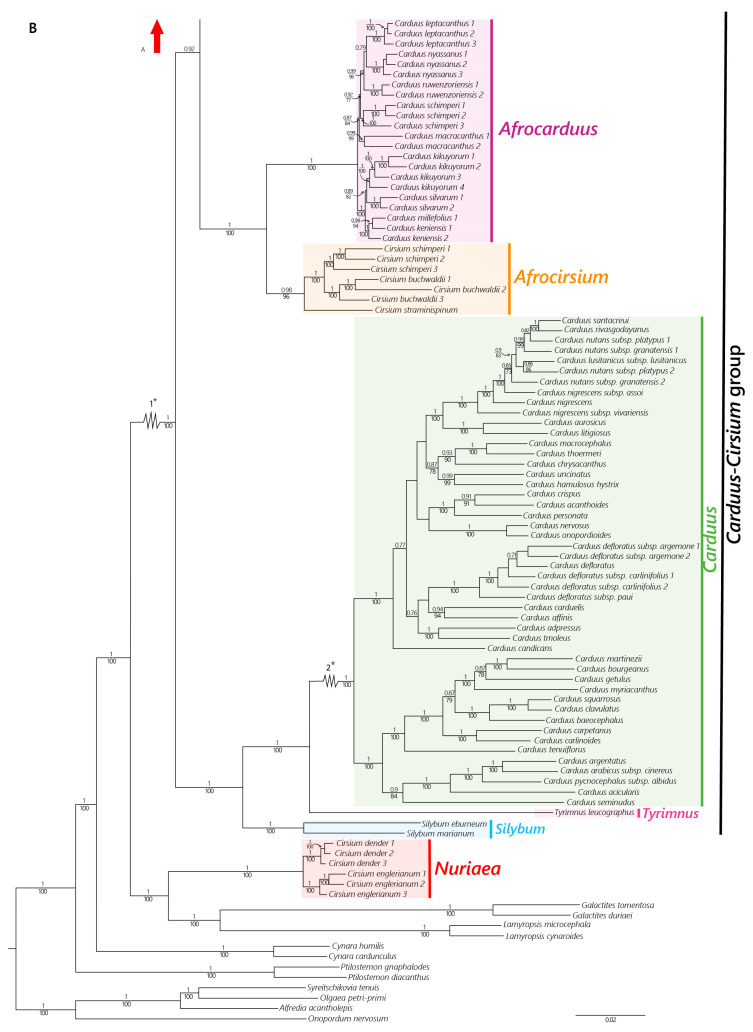
(**A**) Maximum likelihood phylogenetic reconstruction for the subtribe Carduinae obtained with the concatenated nuclear dataset. Values above branches indicate Transfer Expected Bootstrap (TBE) and those below branches indicate Felsenstein’s bootstrap (BS); only values above 70% are shown. (**B**) Maximum likelihood phylogenetic reconstruction for the subtribe Carduinae obtained with the concatenated nuclear dataset. Values above branches indicate Transfer Expected Bootstrap (TBE) and those below branches indicate Felsenstein’s bootstrap (BS); only values above 70% are shown. Branches shortened for fit are indicated on the phylogeny as 1* (50% of reduction) and 2* (30% of reduction).

**Figure 2:**
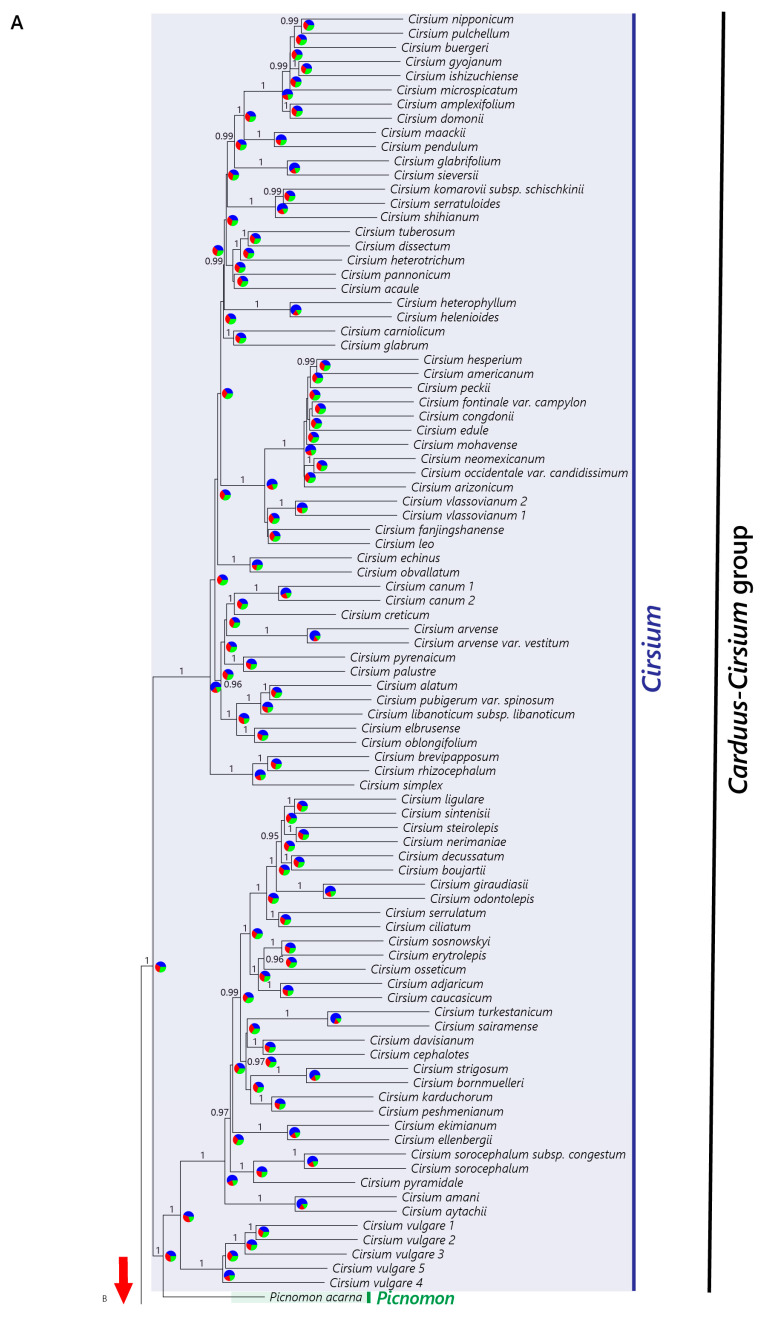

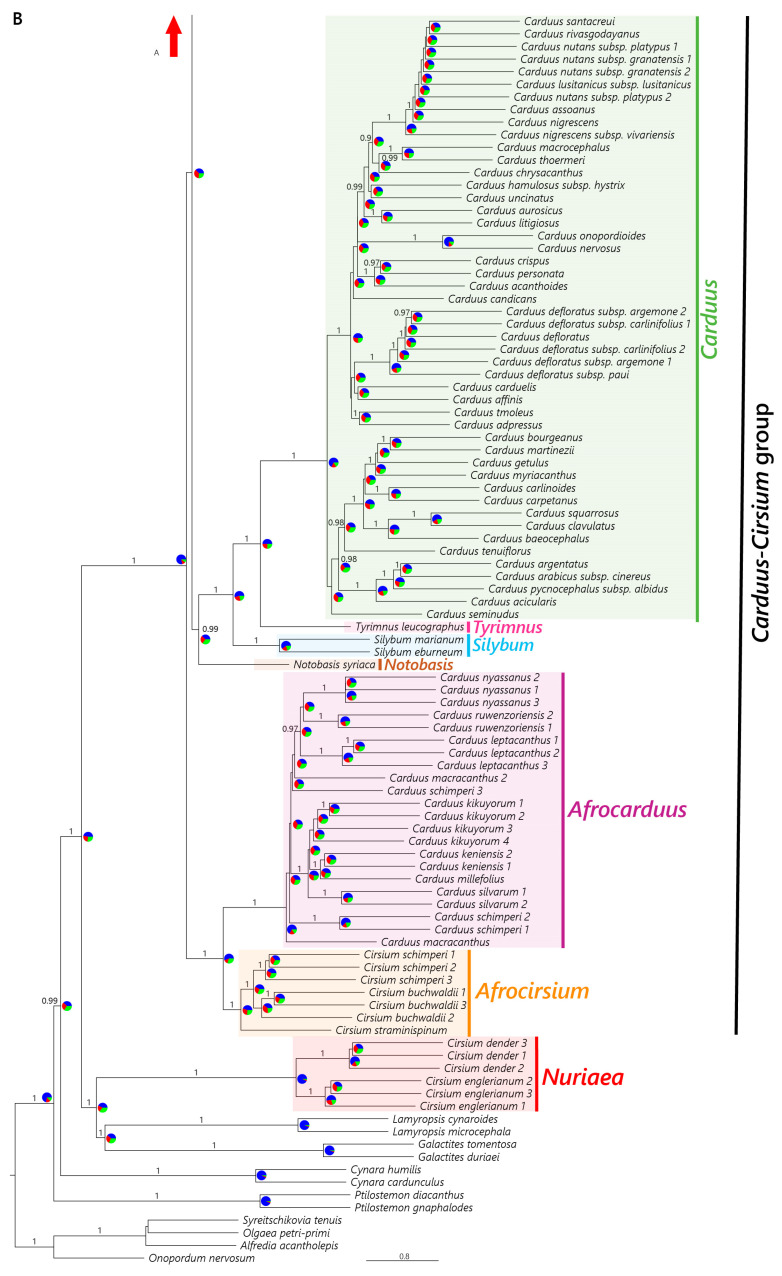
(**A**) Coalescence phylogenetic reconstruction for the subtribe Carduinae obtained with the nuclear dataset. Values above branches indicate Local Posterior Probability (LPP); only values above 0.95 are shown. Pie charts represent the proportion of the gene trees that support the main topology (blue), the first alternative (green) and the second alternative (red) for each node. (**B**) Coalescence phylogenetic reconstruction for the subtribe Carduinae obtained with the nuclear dataset. Values above branches indicate Local Posterior Probability (LPP); only values above 0.95 are shown. Pie charts represent the proportion of the gene trees that support the main topology (blue), the first alternative (green) and the second alternative (red) for each node.

**Figure 3:**
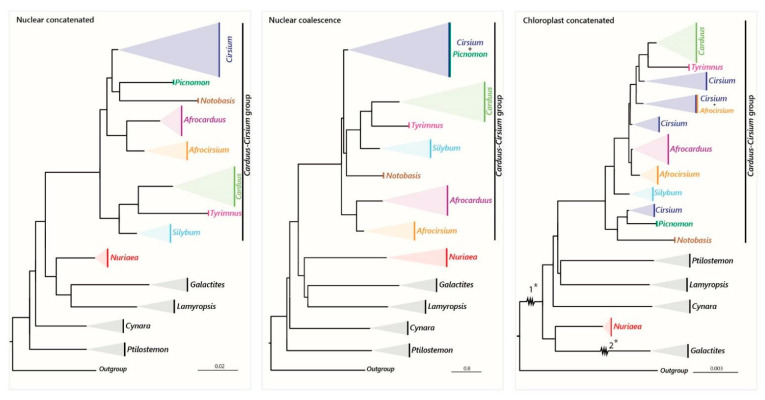
Comparison of the phylogenies obtained with nuclear and plastid data. Clades are shown as collapsed at the generic level, following the classification proposed here. In the chloroplast concatenated tree, branches shortened for fit are indicated on the phylogeny as 1* (60% of reduction) and 2* (30% of reduction).

The concatenated and coalescence approaches yielded phylogenies (Figure 1 and Figure 2) that were highly congruent concerning the relationships between the main clades that fell outside the *Carduus-Cirsium* group, whereas we found several differences within this group (Figure 3). Both phylogenies retrieved subtribe Carduinae as a strongly supported monophyletic group (Figure 1, TBE = 1, BS = 100; Figure 2, LPP = 1). The genus *Carduus* was monophyletic in both analyses (Figure 1, BS = 92, TBE = 0.97, LPP = 1) only when subg. *Afrocarduus* was excluded. The genus *Cirsium* was monophyletic in the concatenated analysis when the African species were excluded. In the coalescence analysis, the African species fell outside *Cirsium*, and the non-African *Cirsium* species plus *Picnomon* formed a monophyletic group. 

The African species traditionally referred to as *Cirsium dender* I.Friis and *C. englerianum* O.Hoffm. formed a monophyletic group (Figure 1, BS = 100, TBE = 1; Figure 2, LPP = 1) outside the *Carduus-Cirsium* clade and as a sister to a clade formed by *Galactites* and *Lamyropsis* (Figure 1, BS = 100, TBE = 1; Figure 2, LPP = 1). The remaining tropical eastern African species traditionally referred to as *Cirsium* formed a monophyletic group (Figure 1, BS = 100, TBE = 1; Figure 2, LPP = 1) as a sister to the clade corresponding to subg. *Afrocarduus* (Figure 1, BS = 100, TBE = 1; Figure 2, LPP = 1). In the concatenated phylogeny (Figure 1), this clade (east African *Cirsium* + subg. *Afrocarduus*) was a sister to *Picnomon+Notobasis*+non-African *Cirsium*, whereas in the coalescence phylogeny (Figure 2), it was a sister to the main clade constituted by all the other lineages of the *Carduus-Cirsium* group.

The genus *Picnomon* was recovered as a sister to *Notobasis* in the concatenated tree, but with low support (Figure 1, BS = 65, TBE = 0.65), forming a lineage that was a sister to *Cirsium s. str.* In contrast, in the coalescence phylogeny, *Picnomon* was nested within the genus *Cirsium* with high support (Figure 2, LPP = 1) and *Notobasis* was recovered with high support (Figure 2, LPP = 0.99) as a sister to the clade constituted by *Silybum, Tyrimnus* and *Carduus s. str*. The genus *Tyrimnus* (Figure 1, BS = 100, TBE = 1; Figure 2, LPP = 1) was recovered as a sister of *Carduus s. str.*, whereas *Silybum* was recovered as a sister to the clade formed by *Tyrimnus* plus *Carduus s. str.* in both phylogenies (Figure 1, BS = 100, TBE = 1; Figure 2, LPP = 1). 

#### 2.2.2. Plastid Dataset

The maximum-likelihood tree inferred from the plastid supermatrix (Figure S2) yielded a highly supported phylogeny except for some shallow nodes within *Cirsium* and *Carduus*. The genera *Cynara*, *Galactites*, *Lamyropsis* and *Ptilostemon* were recovered as monophyletic with high support (all BS = 100, TBE = 1), as was the *Carduus-Cirsium* group (BS = 100, TBE = 1). *Cirsium dender* and *C. englerianum* were recovered outside the *Carduus-Cirsium* group, as in the nuclear analyses (Figure 1 and Figure 2). The genus *Cirsium* was not recovered as monophyletic; it was split into four clades that were nested with other genera of the *Carduus-Cirsium* group. African *Carduus* were recovered as monophyletic (BS = 97, TBE = 0.98) and a sister (BS = 100, TBE = 1) to a clade formed by two species of African *Cirsium* (BS = 100, TBE = 1). In contrast, *Cirsium straminispinum* C.Jeffrey was recovered within one of the *Cirsium* clades. *Silybum* (BS = 100, TBE = 1) and *Carduus* (BS = 100, TBE = 1) were recovered as monophyletic when African *Carduus* were excluded. *Tyrimnus* (BS = 100, TBE = 1) was recovered as a sister to the latter, as in the two nuclear-based phylogenies (Figure 1 and Figure 2). *Notobasis* and *Picnomon* were recovered as successive sister lineages to one of the *Cirsium* clades, in both cases with full support (BS = 100, TBE = 1).

## 3. Materials and Methods

### 3.1. Taxon Sampling

A total of 190 samples (from both herbarium specimens and specific field campaigns) of 159 species representing all the genera of subtribe Carduinae as defined by Herrando-Moraira et al. (2019) [32] were selected for our study (Table S1). Samples of *Cirsium* and *Carduus* were chosen to cover both their taxonomic diversity and subcosmopolitan distribution. Especially for the Afromontane and Afroalpine taxa, we sampled broadly to cover morphological heterogeneity and possible misleading taxonomical determinations. We also included two species of *Silybum*; the monotypic genera *Notobasis*, *Picnomon* and *Tyrimnus*; and two species of each of the other genera of Carduinae: *Cynara* L., *Galactites* Moench, *Lamyropsis* (Kharadze) Dittrich and *Ptilostemon* Cass. Finally, four species of Onopordinae (sister to Carduinae) were included as outgroup taxa based on previous phylogenetic results at the tribal level [32]. For eleven species, we used the raw reads previously obtained in a latter study, whereas all other sequences were newly generated either from herbarium specimens or fresh material collected in the field and preserved in silica gel. 

### 3.2. DNA Extraction, Library, Capture and Sequencing

For each sample, 1–50 mg of dried plant material was ground. DNA was extracted using an E.N.Z.A^®^ SP Plant DNA Kit (Omega Bio-Tek Inc., Norcross, GA, USA) following the manufacturer’s instructions. DNA was measured using a Qubit™ Flex Fluorometer (Thermo Scientific, Waltham, MA, USA) and sheared (0.2–1 µg in 50 µL) using a Qsonica Q800R3 Sonicator (Qsonica LLC, Newtown, CT, USA) at 20% amplitude for 45 sec to 10 min at 4 °C to obtain fragments of 300–400 bp. Fragment length was checked with electrophoresis in 1.2% agarose gels. Libraries were prepared using an NEBNext Ultra II DNA Library Prep Kit for Illumina^®^ (New England Biolabs, Ipswich, MA, USA) from 25 to 45 µL of sonicated DNA using half of the volumes stipulated by the manufacturer and fifteen cycles of PCR amplification. Libraries were barcoded with single or dual index primers, NEBNext^®^ Multiplex Oligos for Illumina^®^. Libraries were quantified with Qubit and those with more than 17 ng of DNA were pooled to a max. of 10 samples and 2 µg of total DNA (around 250 ng for each sample). Subsequently, pools were evaporated or filled with water to 7 µL of volume to execute the target-enrichment protocol of [36] using a Microarray MyBaits COS kit (Daicel Arbor Biosciences, Ann Arbor, MI, USA). To sequence plastid DNA, 40% of the DNA from the libraries previous to the target enrichment step was added to each pool. Pools were sequenced (PE 150 bp) using HiSeq 2500 and HiSeq X. For some samples, the DNA was extracted and sent to Daicel Arbor Biosciences for library construction, target-enrichment capture and posterior sequencing on an Illumina NovaSeq (PE 150 bp).

### 3.3. Molecular Data Processing 

HybPhyloMaker [37] scripts available at https://github.com/tomas-fer/HybPhyloMaker (accessed on 4 November 2021), a bioinformatic pipeline specially developed to process Hyb-Seq data, were used to process raw sequence reads and conduct phylogenetic inference analyses, in combination with ParalogWizard [38]. ParalogWizard is a workflow developed to detect paralogous sequences and separate them into different alignments based on sequence similarity. Thus, this procedure allows for obtaining two or more orthologous alignments from an alignment that included paralogous sequences. This approach avoids losing potential informative sites in paralogous loci and reduces gene tree discordance in recent and rapidly radiated groups [39]. 

Raw reads were cleaned using Trimmomatic v.0.32 [40] as implemented in HybPhyloMaker. To obtain the targeted nuclear data, the Compositae1061 probe set [36] was used for initial read mapping using BWA [41] and SPAdes scripts [42] as implemented in ParalogWizard. Pairwise sequence divergence was calculated to identify paralogues, resulting in two peaks: the first represented putative allelic variation, and the second represented highly divergent sequences corresponding to putative paralogues. The divergence value of the second peak was used as the threshold value to identify putative paralogous sequences and separate them into orthologous matrices, which were aligned using MAFFT v.7.029 [43]. The obtained alignments were processed with HybPhyloMaker (scripts 5 and 5b) to reduce missing data. We removed sequences missing more than 70% of the total locus length and loci for which less than 75% of the samples were represented. 

To obtain plastid sequences, we created a reference file from the complete plastome sequence of *Cirsium arvense* (NCBI Accession number: KY562583) including a total of 201 regions: 86 genes (coding regions + introns), 8 rRNAs, 37 tRNAs and 70 intergenic regions extracted using Artemis v.18.2 [44]. Quality-trimmed reads were mapped using this reference and the BWA method as implemented in HybPhyloMaker. The subsequent match of the mapped sequences with the probes, alignments and processing of the missing data was carried out using HybPhyloMaker with the same criteria as for the nuclear data (<70% of missing data and a >75% sample presence per gene).

### 3.4. Phylogenetic Analyses

We used both concatenation and coalescence approaches to infer phylogenetic relationships. In the concatenation approach, a nuclear-based phylogenetic tree was inferred based on a single supermatrix of all nuclear loci retrieved; the same process was repeated with all plastid regions combined in a second supermatrix of all chloroplastic regions retrieved. The coalescence approach to generate a species tree was only applied to the nuclear dataset since all chloroplast regions are assumed to be linked.

With each supermatrix, nuclear and chloroplast, phylogenetic inference analyses under maximum likelihood were carried out using RaxML-NG [45] with 20 independent tree searches and applying the best-fit model for each partition (one for each locus) previously determined using Modeltest-NG [46]. To measure branch support, Felsenstein’s Bootstrap BS [47] and the Transfer Bootstrap Expectation TBE [48] were calculated using RaxML-NG and applying the bootstopping criterion with the default to determine the sufficient number of replicates, cf. [49,50]. We included TBE in addition to BS because it has been reported that the former is more suitable for studies with large datasets. In TBE, the presence of inferred branches in bootstrap trees is measured with a gradual ‘transfer’ distance, while in BS, it is based on binary presence/absence [48]. As a result, when using a large number of taxa, it becomes harder to resample clades exactly as in the original tree, causing an underestimation in large clades and obtaining significantly lower values of BS at deep nodes [51]. For both metrics, the value of 70% was chosen as the threshold indicating supported branches.

For the coalescence approach, nuclear gene trees were inferred using RaxML v.8.2.12 [52] with a bootstrap resampling of 100 replicates. Based on these gene trees, a species tree was obtained using ASTRAL-III v.5.7.8 [53]. Support values were calculated using local posterior probabilities LPP [54], and branches were considered well-supported at LPP ≥ 0.95. To detect the degree of incongruence between gene trees, a quartet-based method analysis was run with the −t 8 option of ASTRAL-III, which allows for identifying the percentage of genes that support alternative topologies for each node [55].

### 3.5. Morphological Examination

Both before and after the phylogenetic analyses, a morphological study of the genera was undertaken. We studied a total of 74 herbarium sheets from herbaria (Institut Botànic de Barcelona (BC), Meise Botanic Garden (BR), Muséum National d’Histoire Naturelle (P) and Naturalis Biodiversity Center (WAG)), and material collected in the field. The complete list of specimens examined is provided in the description of the new genera. The characters we examined (Table S2) were those considered relevant by Kazmi (1963) [29]. 

## 4. Discussion

### 4.1. Utility of Hyb-Seq and Incongruence between the Phylogenies

Our analyses yielded highly supported phylogenetic reconstructions, reaffirming the effectiveness of NGS combined with the target-capture methodology proposed by Mandel et al. (2014) [36] in resolving relationships in Compositae at different taxonomical levels [32]. Based on the extensive nuclear dataset comprising 986 loci, we successfully constructed a phylogeny for Carduinae with strongly supported groups. 

Concerning the relationships among the main clades, we found some incongruence between the nuclear concatenated and coalescence phylogenies (Figure 1 and Figure 2). In the concatenated tree, the East African clade was a sister to a clade consisting of the remaining *Cirsium* species plus *Notobasis* and *Picnomon*, whereas in the coalescence tree, the East African clade was a sister to the *Carduus-Cirsium* clade (Figure 2). Such inconsistency may be attributable to an incongruent phylogenetic signal among genes due to incomplete lineage sorting (ILS), which has been shown to be problematic in concatenated approaches [56,57]. This explanation is supported by our quartet support analysis (Figure 2), which indicated nearly equal proportions of genes supporting alternative topologies for the nodes concerned. Such results have been suggested to indicate high levels of ILS [55]. 

The plastome analysis yielded a highly supported tree also showing the monophyly of the *Carduus-Cirsium* group, and suggests that the African species of this group represent three independent evolutionary lineages (except for *Cirsium straminispinum*; see details below). The main incongruence between the plastid and nuclear trees was found within the *Carduus-Cirsium* group, concerning *Cirsium* taxa (Figure 3). In the nuclear trees, the non-African *Cirsium* species were either recovered as a separate clade (Figure 1) or nested with *Picnomon* (Figure 2). In contrast, the plastid tree split *Cirsium* into four clades: one close to *Picnomon* and *Notobasis* and three forming a graded clade in which *Carduus + Tyrimnus* are nested (Figure 3). Such incongruences may result from several non-exclusive factors that promote unstable phylogenetic relationships, such as rapid radiations, ILS and hybridization [58,59,60]. Indeed, hybridization or introgression have been reported multiple times in the evolutionary history of the tribe Cardueae, e.g., [61].

### 4.2. The African Carduus and Cirsium

The monophyly we inferred for the African species traditionally referred to as *Carduus* (Figure 1, Figure 2 and Figure S2) is consistent with previous morphological [17,29] and molecular studies [19,25]. The main difference between Eurasian and African *Carduus* (subg. *Afrocarduus*) is found in the morphology of the achene. Häffner (2000) [17] was the first to point out that these taxa could constitute a lineage more related to *Cirsium* than to *Carduus* based on the shape of the dorsal corolla lobe epidermis (straight as in *Cirsium*, not undulate as in *Carduus*), indument of the stamen filaments (glabrous as in *Cirsium*, pilose as in *Carduus*) and characters of the achene pericarp (with four blunt ribs and persisting at maturity as in *Cirsium*, not with 10–15 longitudinal stripes and disintegrating at maturity as in *Carduus*). Their chromosome numbers are also similar: *Carduus* usually has *n* = 9, and both *Carduus* subg. *Afrocarduus* and *Cirsium* have *n* = 16–17 (17). Our examination of 74 herbarium specimens confirmed all reported morphological differences. The reason for the traditional placement of these African species in *Carduus* is that *Carduus* and subg. *Afrocarduus* always lack plumose pappus, which is found in *Cirsium s. str.* and African *Cirsium*. Our results thus demonstrate that the African *Carduus* form a distinct evolutionary lineage that does not belong neither to *Cirsium* nor to *Carduus*, that it can be diagnosed using several morphological characters and that it is endemic to the tropical mountains of eastern Africa. Thus, a new classification that accurately represents the phylogenetic history of the group is needed. Here, we propose a new genus using the name provided by Kazmi (1963) [29] for the subgenus: *Afrocarduus* (Kazmi) Garcia-Jacas, Moreyra & Susanna, gen. et stat. nov. Concerning the number of species—nine (Jeffrey, 1968 [31]) vs. 22 (Kazmi, 1963 [29])—our results and our ongoing research (Moreyra et al., unpubl. data) seem to suggest a number close to that suggested by Kazmi (1963) [29]. For example, *Carduus kikuyorum* R.E.Fr. was considered a subspecies of *Carduus nyassanus* R.E.Fr. by Jeffrey (1968), but it should be considered an independent species as proposed by Kazmi (1963) [29], since they are independent evolutionary lineages, according to our phylogenies (Figure 1 and Figure 2). A complete taxonomic treatment for *Afrocarduus* is under preparation and will be published in a separate work.

Three of the African species traditionally referred to as *Cirsium* (Figure 1 and Figure 2; *C. buchwaldii* O.Hoffm., *C. schimperi* (Vatke) C.Jeffrey and *C. straminispinum*) were recovered together as a clade sister to *Afrocarduus* with the nuclear dataset. *Cirsium straminispinum* was recovered within the *Cirsium* clade in the plastome analysis (Figure S2), and we consider it likely that this may be a signal of a hybrid origin of this species [25]. These three species have the typical plumose pappus of *Cirsium* [14], but they also show distinctive characters such as phyllaries with well-developed pectinate appendages, which are absent in *Cirsium* and in all other genera in the *Carduus-Cirsium* group. We are aware of the weakness of suggesting a new taxon based on a single morphological character, especially given the difficulties to differentiate groups in the subtribe Carduinae [18]. However, we consider the presence of a unique diagnostic morphological character combined with strong evidence of being an independent evolutionary lineage to be sufficient to propose a new genus, *Afrocirsium* Calleja, Garcia-Jacas, Moreyra & Susanna, gen. nov., that is a sister to *Afrocarduus* in all the analyses.

Finally, two Ethiopian species traditionally referred to as *Cirsium*, *C. dender* and *C. englerianum* were recovered as a monophyletic group falling outside the *Carduus-Cirsium* clade, as a sister to *Galactites* and *Lamyropsis* (Figure 1 and Figure 2) or a sister to *Galactites* (Figure S2). These species share the plumose pappus with *Cirsium s. str.* and *Afrocirsium*. However, as stressed by Friis (1975) [62], the two species differ conspicuously from *Cirsium/Afrocirsium* by their large capitula (4–7 cm) that resemble those of *Cynara* [63]; a strong (>2 mm width) and long thorn (>30 mm) on their basal leaf lobes; and their large size (2–5 m). We therefore propose a new genus, *Nuriaea* (Friis) Susanna, Calleja & Moreyra, comb. nov., to accommodate these two species. 

### 4.3. The Carduus-Cirsium Group

The *Carduus-Cirsium* group was recovered as monophyletic, and after segregating the new genera *Afrocarduus* and *Afrocirsium*, it contains eight genera: *Afrocarduus, Afrocirsium, Carduus*, *Cirsium*, *Notobasis*, *Picnomon*, *Silybum* and *Tyrimnus* (Figure 1 and Figure 2). The main incongruence between the coalescence and concatenation trees concerns the positions of the monotypic genera *Picnomon* and *Notobasis*. *Notobasis* was recovered as a sister to *Picnomon* (Figure 1), to *Carduus* + *Tyrimnus* + *Silybum* (Figure 2) or to *Picnomon* + *Cirsium* (Figure S2). These inconsistencies could result from ILS and/or hybridization (as discussed above). In all analyses, *Notobasis* was recovered as an independent lineage with a long branch length, and we, therefore, suggest maintaining its generic status. We also tentatively favor maintaining *Picnomon* as a distinct genus because (1) it was recovered as an independent lineage in the nuclear and plastid trees obtained under concatenation; (2) in the coalescence tree, it was instead recovered as a sister to one of the two main clades of *Cirsium*, but both the node leading to *Picnomon* and the preceding node had similar proportions of genes supporting alternative topologies (Figure 2). A final decision to confirm the appropriateness of this choice should await further research including more individuals.

*The genus *Cirsium. The status of *Cirsium* (for all non-African species) as a single entity has often been questioned, and the need for further research using more accurate methods to make taxonomical decisions has been pointed out [19]. Recently, the authors of [20] split *Cirsium* into four genera (*Cirsium s. str.*, *Lophiolepis*, *Epitrachys* and *Lophiocirsium*). This work encompasses a remarkable number of species (*n* = 225), yet it is based only on two nuclear and five plastid markers that have proven to be insufficient for phylogenetic resolution in this genus [19]. Moreover, according to the Supplementary Material [20], Table S2, the matrix lacks almost 50% of sequences (880 out of 1785). Moreover, the published phylogeny fails to support the classification proposed since *Cirsium s. str* is not recovered as monophyletic as the entire genus *Carduus* was nested within *Cirsium s. str.* In addition, two species of African *Carduus*, now *Afrocarduus*, are recovered within *Cirsium s. str.* [20], Figure S1, whereas our study reveals that African species of *Carduus*, now *Afrocarduus*, are a group evolutionary independent from *Cirsium* (Figure 1, Figure 2 and Figure 3). 

Interestingly, our plastid phylogeny agrees with the polyphyletic status of *Cirsium*, which was split into the same clades as in [20]’s phylogeny, and *Carduus* was nested within one of them. It is therefore likely that the results of [20] are strongly influenced by the plastid markers. In contrast, the classification in [20], Figure S1, is not supported by our nuclear phylogenies, where all species we included from sections *Eriolepis* (Cass.) Dumort. and *Cirsium* (according to [20]) are recovered as closely related and seem to share the same origin (Figure 1 and Figure 2). Thus, our analyses based on nuclear data succeeded in recovering the natural groups within the group *Carduus-Cirsium* after segregating the African species, with high definition and strong support, probably thanks to our much larger molecular dataset obtained with the Hyb-Seq approach. In our opinion, this classification is the most conservative and morphologically consistent [14]. Furthermore, given that *Cirsium* comprises more than 450 species with many practical applications, see [19], our conservative classification maintaining *Cirsium* as a single genus is also the most robust and operational one, because it avoids the inflation of hundreds of new nomenclatural combinations that would increase the already voluminous synonymy of *Cirsium*. 

Additionally, hybrids between the two largest new genera proposed in [20] are extremely frequent [20,64]. Hybridization between species and supra-specific entities constitutes an evolutionary driver and also a taxonomic and systematic challenge [65]. This fact might also support that *Cirsium* should be treated as a single genus [19] until new studies covering the entire genus (diversity and distribution) are published. Meanwhile, the two subgenera, *Cirsium* and *Lophiolepis* Cass., can be distinguished by the absence or presence of setae on the upper leaf surface, respectively. A recent study (which partially covers the diversity and distribution) has provided some other characters that differ between the two subgenera: the monoploid genome size, genomic GC content and size of guard cells and achenes [64].

Finally, one species of *Cirsium s. str.* (*Cirsium vulgare* (Savi) Ten.) was recovered within sect. *Eriolepis* in our nuclear analyses (Figure 1 and Figure 2). In our preliminary nuclear analyses, *Cirsium vulgare* had an unstable position between the two main clades of *Cirsium* (trees not shown), possibly because of a hybrid origin. This hypothesis is supported by the incongruence we detected between the plastome and nuclear phylogenies: in the plastome tree (Figure S2), *Cirsium vulgare* was recovered within *Cirsium s. str.*, whereas it was recovered as a sister to a clade formed by all taxa of sect. *Eriolepis* in the nuclear trees (Figure 1 and Figure 2). Similar results have been obtained for *Cirsium italicum* DC. [19], which could be of a hybrid origin [20,65]. New analyses including a wider taxonomic sampling of the genus *Cirsium* are necessary to address a better infrageneric classification for *Cirsium* (Moreyra et al. in prep.).

*The genus* Carduus. The genus *Carduus s. str.* was recovered as a well-defined group in all our analyses (Figure 1, Figure 2 and Figure S2). *Tyrimnus* was recovered as a sister to *Carduus*, which is consistent with morphological characters. Originally, *Tyrimnus* was described as *Carduus leucographus* L. due to its morphological resemblance to *Carduus.* However, *Tyrimnus* can be well differentiated from *Carduus* from its s-shaped pappus bristles (straight in *Carduus*). We found the two species of *Silybum* to be a sister to *Tyrimnus* + *Carduus*. Within *Carduus*, we retrieved two clades that might deserve subgeneric status, but a final decision must await wider sampling and further analyses. 

## 5. Taxonomic Proposal

***Afrocarduus* (Kazmi) Garcia-Jacas, Moreyra & Susanna, gen. et stat nov.**
≡ *Carduus* subgenus *Afrocarduus* Kazmi, Mitt. Bot. Staatssamml. München 5: 139 (1963) [basionym].
Spiny perennial herbs, sometimes rosulate and acaulescent, more often caulescent to 2.5 m high. Stems usually winged or interruptedly winged, fistulose. Leaves very variable, from narrowly lanceolate to oval–lanceolate or elliptic, dentate to pinnatisect, spiny specially on lobes and teeth, glabrous or sparsely arachnoid–pilose above, more densely pilose below especially on nerves, often with septate hairs; nerves prominent. Capitula globose or campanulate, terminal, solitary or many-clustered in the center of rosettes in acaulescent species, homogamous, 0.5–3 cm wide. Involucral bracts glabrous or arachnoid–pilose, sometimes lanuginose; margin minutely denticulate of entire, scariose, glabrous or pilose with septate hairs, without appendages; apical spine always present, 1 to 20 mm long. Florets white, pink or purple; anther filaments papillose with very short papillae to 0.1 mm. Achenes ovate, laterally compressed, glabrous, smooth or somewhat striate, 3–9 mm long, with a short apical coronule; apical caruncle absent. Pappus to 55 mm long; setae scabrid-short barbellate. Type: *Afrocarduus leptacanthus* (Fresen.) Garcia-Jacas, Moreyra & Susanna.
***Afrocarduus afromontanus*** **(R.E.Fr.) Garcia-Jacas, Moreyra & Susanna, comb. nov.**
≡ *Carduus afromontanus* R.E.Fr., Acta Horti Berg. 8: 21 (1925) [basionym].
**Examined material: Kenya**: South Kinangop, The Elephant Mt. alt. 2700 m, 6.5.1968, *O.M.Mwangangi 1003* (BR0000016058613); Mt. Kenya (versant ouest), alt. 3300 m, 02.1912, *C.Alluaud KE204* (MNHN-P-P0010072).
***Afrocarduus keniensis*** **(R.E.Fr.) Garcia-Jacas, Moreyra & Susanna, comb. nov.**
≡ *Carduus keniensis* R.E.Fr., Acta Horti Berg. 8: 31 (1925) [basionym].
**Examined material: Ethiopia: Kenya:** Mt. Elgon, 06.12.1920, *G.Lindblom s.n.* (S! [photo]). East side of Mt. Kenya, alt. 3078 m, 05.15.1926, *J.P.Chapin 48* (BR0000016058637); Mt. Kenya (Versant Ouest), alt. 3200–3300 m, 02.06.1912, *K.E. (?) 214* (MNHN-P-P0010092). **Tanzania:** Kilimandjaro, alt. 3000–4000 m, *P.E.Janssens s.n*. (BR0000016058644); Kilimandjaro, alt. 3580 m, 03.07.1934, *H.J.E.Schlieben 4912* (BR0000016058675); Kilimandscharo, SO seite, alt. 3580 m, 03.07.1934, *H.J.Schilieben 4912* (MNHN-P-P0010088).
***Afrocarduus kikuyorum*** **(R.E.Fr.) Garcia-Jacas, Moreyra & Susanna, comb. nov.**
≡ *Carduus kikuyorum* R.E.Fr., Acta Horti Berg. 8: 24 (1925) [basionym] ≡ *Carduus nyassanus subsp. kikuyorum* (R.E.Fr.) C.Jeffrey, Fl. Trop. E. Africa 49 (1969).
**Examined material: Kenya:** Samburu, Mt. Nyiru, Mario Forest Zone, alt. 2500 m, 03.30.1995, *B.Bytebier 232* (BR0000016059610). **Rwanda:** N.-Rwanda, O.-flank van de Muhavura, alt. 3100 m, 02.19.1972, P. N.-Rwanda, *O.-flank van de Muhavura 9457* (BR0000021711428); Northern Province, Volcanoes National Park, Muhavura volcano, *M.Galbany-Casals 2779, J.A.Calleja & M.Kandziora* (BC)*;* Northern Province, Volcanoes National Park, Muhavura volcano, *M.Galbany-Casals 2790, J.A.Calleja & M.Kandziora* (M.Galbany pers.herb.). **Tanzania:** Kilimanjaro, alt. 1800 m, 12.16.1933, H.J.E. Schilieben 4361, (BR0000016060241); Ngorongoro conservation area, Empakaai Crater, top of the south rim, alt. 2900 m, Eastern exposure, 02.28.1973, *G.W.Frame P21* (BR0000016060265). **Uganda:** Western Province, Kigezi Dist. Virunga-Kette, Sattel zwischen Muhavura und Mgahinga, alt. 3000 m, 11.14.1954, *H.U.Stauffer 771* (BR0000016060197).
***Afrocarduus leptacanthus* (Fresen.) Garcia-Jacas, Moreyra & Susanna, comb. nov.**
≡ *Carduus leptacanthus* Fresen., Mus. Senckenberg. 3(1): 70 (1839) [basionym]
= *Carduus abyssinicus* Sch.Bip., Linnaea 19(3): 332 (1846).
**Examined material: Congo:** Parc National Albert, entre Kakalali et Butahu, le long du chemin-limite du P.N.A. (Sousd. du Ruwenz.), Congo Belge, alt. approx. 1650 m, 08.05.1952, *H.Fredericq 7857* (BR0000016059047). **Ethiopia:** sine loc., *E.Rüppell s.n.*, 06.01.1832 (FR! [photo]). **Kenya:** Landiani. Mau escarpment, alt. 2500 m, 10.1903, *C.Alluaud KE80* (MNHN-P-P0010115). **Rwanda:** Wisumo, Prefecture: Kibuye, alt. 2200 m, 03.16.1973, *G.Troupin 14755* (BR0000016059368); N.-Rwanda, terr. Ruhengeri, Kinigi, Rops-plantage, alt. approx. 2300 m, 02.24.1972, *P.Van der Veken 9539* (BR0000016059375); Northern Province, Volcanoes National Park, Mount Gahinga, cultivated lands outside the park limit, 02.08.2022, *M.Galbany-Casals 2756, J.A.Calleja & M.Kandziora* (M.Galbany pers. herb.). **Tanzania:** Ruvuma region Kitulo, 10.18.1969, *J.Prins-Lampert 576* (WAG.1744238).
***Afrocarduus macracanthus* (Kazmi) Garcia-Jacas, Moreyra & Susanna, comb. nov.**
≡ *Carduus macracanthus* Kazmi, Mitt. Bot. Staatssamml. München 5: 164 (1963) [basionym].
**Examined material: Ethiopia:** Bale prov., Bale Mts National Park, Garba Goracha, in steep slope just S of the camp site, alt. 3950 m, 11.02.1973, *K.O.Hedberg 5623* (WAG.1361919); Mt. Boruluccu, along the road to Ticcio, about 30 km SE of Asella, alt. 4000 m, 12.06.1965. *W.J.J.O.Wilde & B.E.E. De Wilde-Duyfjes 9187* (BR0000016059559); Mt. Boruluccu, along the road to Ticcio, about 30 km SE of Asella, alt. 4000 m, 12.06.1965, *W.J.J.O.De Wilde & B.E.E.Wilde-Duyfjes* (MNHN-P-P0010116).
***Afrocarduus millefolius*** **(R.E.Fr.) Garcia-Jacas, Moreyra & Susanna, comb. nov.**
≡ *Carduus millefolius* R.E.Fr., Acta Horti Berg. 8: 34 (1925) [basionym].
**Examined material: Kenya:** Western slopes of Mt. Kenya, along the trail from West Kenia Forest Station to summit, British East Africa, alt. 3630 m, 09.21–27.1909, E.A. Mearns, (BR0000016059566); Fort Jerusalem, Aberdares National Park on N. Kinangop-Nyeri road, alt. 3200 m, 7.30.1960, *E.Polhill 228*, (BR0000016059573); Mt. Kinangop, alt. 2800 m, 02.18.1912., *C.Alluaud ke 268* (MNHN-P-P0010124).
***Afrocarduus nyassanus* (S.Moore) Garcia-Jacas, Moreyra & Susanna, comb. nov.**
≡ *Carduus leptacanthus* var. *nyassanus* S.Moore, Journ. Linn. Soc. Bot., 37: 326 (1906) [basionym] ≡ *Carduus nyassanus* (S.Moore) R.E.Fr., Acta Horti Berg. 8: 25 (1925).
**Examined material: Congo:** Bord marécageux d’une rivière, Près de Kasiki (Plateau des Marungu, Katanga), alt. 2000 m, 11.26.1969, *S.Lisowski, F.Malaisse & J.Symoens 8378* (BR0000016059948). **Malawi:** Zomba plateau, Chingwe’s Hole area, alt. 1800 m, 11.20.1981, *J.D.Chapman & E.J.Tawakali 5998* (BR0000016059641). **Rwanda:** Northern Province, Volcanoes National Park, Mount Gahinga, 02.08.2022, *M.Galbany-Casals, J.A.Calleja & M.Kandziora 2765.*
***Afrocarduus ruwenzoriensis*** **(S.Moore) Garcia-Jacas, Moreyra & Susanna, comb. nov.**
≡ *Carduus ruwenzoriensis* S.Moore, J. Linn. Soc., Bot. 35: 364 (1902) [basionym].
**Examined material:** **Congo**: Ruisseau affluent de la Mososa, (à l’Est de Mahungu), alt. 3180 m, 05.29.1953, *F. in De Witte 9134* (BR0000005891160).
***Afrocarduus schimperi* (Sch.Bip.) Garcia-Jacas, Moreyra & Susanna, comb. nov.**
≡ *Carduus schimperi* Sch.Bip., Linnaea 19 (3): 334 (1846) [basionym].
**Examined material:** **Congo**: Mts. S.W. of Lemera, lower Ruzizi Valley, Kivu district, alt. 2830 m, 07.17.1927, *J.Chapin 515* (BR0000016059450); Mont Muki, Prairie, alt. 3050 m, 07.27.1955, *U.J.Kinet 5* (BR0000016059481); Munt Muhende, 03.1982, *H.Scaetta 2424* (BR0000016059504); Mont Muhende, *H.Scaetta 2424* (BR0000016059511); Sur les crêtes rocheuses du Mt. Kyanza au Sud Ouest du Lac Lungwe, alt. 2700 m. 08.01.1959, *A.Michelson 1078* (BR0000016059498). **Ethiopia:** Kaffa: SE of Folla, some 15 km N of Ghibe bridge (on Addis-Jimma road), alt. 2000–2100 m, 12.02.1970, *I.Friis, A.Hunde, K.Jacobsen 548* (WAG.1361800); Gojam, near Debre Markos, on the road to Elias, alt. 2175 m, 12.22.1972, *C.J.P.Seegeler 2968* (WAG.1361869); Gondar, Semiam Mountains, small valley running down to main valley between Geech and Ambaras, alt. 3360 m, 09.19.1969, *M.G.G.Gilbert 105* (WAG.1361871); about 10 km SE of Hagere Selam, SE of Wondo, alt. approx. 3000 m, 03.13.1966, *W.J.J.O de Wilde & B.E.E.Wilde-Duyfjes 10299* (WAG.1361877); Road Wondo-Agere Selam, 17 km, from Wondo, alt. approx. 2450 m, 11.17.1967, *E.Westphal & J.Westphal-Stevels 2675* (BR0000016060616); Mt. Entotto, about 5 km N. of Addis Ababa, alt. approx. 2600 m, 02.12.1966, *E.Westphal & J.Westphal-Stevels 9974* (BR0000016060623); Kaffa prov. S.E. of Folla, some 15 km N. of Ghibe bridge, on Addis-Jimma road, alt. 2000–2100 m, 12.02.1970, *I.Friis 548* (BR0000016060630); Begemder Prov. Semian Mountains, small valley running to main valley, between Geech and Ambaras, alt. approx. 3360 m, 09.19.1969, *J.J.F.E. de Wilde & M.G.Gilbert 105* (BR0000021711459); Begemder Prov. Semian Mountains, small valley running to main valley, between Geech and Ambaras, alt. approx. 3360 m, 09.19.1969, *J.J.F.E. de Wilde & M.G.Gilbert 105* (BR0000021711459); Mt Wanchi, near rim of crater, alt. 3100 m, 09.20.2003, *C.C.H.Jongkind 5974* (WAG.1362060); Col de Mororo, prov. Bale, alt. 3200 m, 05.16.1970, *J.L.Guillaumet 2674* (MNHN-P-P00931227). **Kenya:** Nanyuki distr. Endarasha ranch near Sirimun R., alt. 2000 m, 08.09.1965, *J.B. Gillett 16827* (MNHN-P-P0010076); S. Nyanza, Kisii district (k5), alt. 1930 m, near Ramasha, 01.14.1978, *A.C.Plaizier 483* (WAG.1361797); Ol’Pusimoru sawmill about 9 miles from Olokurto, alt. 2600 m, 05.20.1961, *P.E.Glover, Gwynne & Samuel 1339* (BR0000016060678). **Tanzania:** Masai district, 15 km from Lolionto on Marok road, 11.09.1974, *J.B. Gillet 16333* (BR0000016060708); on way to Aiteho (illegible), Hubulu dist., alt. 1828 m, 08.31.1932, *B.D.Burtt 4268* (BR0000016060685).
***Afrocarduus silvarum*** **(R.E.Fr.) Garcia-Jacas, Moreyra & Susanna, comb. nov.**
≡ *Carduus silvarum* R.E.Fr., Acta Horti Berg. 8: 22 (1925) [basionym].
**Examined material: Kenya:** Upper Imenti Forest, within Meru Municipality boundary, alt. 1740 m, 05.28–29.1974, *R.B. & A.J.Faden 74/914* (BR0000016059528); 10 km E of Kieni, alt. 2100 m, 06.17.1986, *K.H.J.Beentje 2938* (WAG.1362065). Uganda: British Uganda, Station Lamuru (Buschiges Hochland), alt. 3000, 07.11.1909, *G.Schefflet 330* (MNHN-P-P0010145).
A complete taxonomic and nomenclatural revision of *Afrocarduus* is in preparation and will be published as a separate work.
***Afrocirsium* Calleja, Garcia-Jacas, Moreyra & Susanna, gen. nov.**
Spiny perennial herbs, caulescent, 0.50 to 2.5 m high. Stems unwinged, fistulose. Leaves lanceolate, shortly decurrent, entire, lobate or pinnatisect with spiny lobes, glabrescent above, white–tomentose below, usually with reticulate nerves. Capitula solitary or clustered, campanulate, 1–3 cm wide, homogamous. Involucral bracts ovate or lanceolate; margin scariose, denticulate, the outer ones with a fimbriate, black appendix; apical spine short, to 3 mm. Florets pink or purple; anther filaments papillose with very short papillae to 0.1 mm. Achenes ovate, laterally compressed, glabrous, smooth or somewhat striate, 4–7 mm long, with a short apical coronule; apical caruncle absent. Pappus to 12 mm long; setae plumose. Type: *Afrocirsium schimperi* (Vatke) Calleja, Garcia-Jacas, Moreyra & Susanna, comb. nov.
***Afrocirsium buchwaldii*** **(O.Hoffm.) Calleja, Garcia-Jacas, Moreyra & Susanna, comb. nov.**
≡ *Cirsium buchwaldii* O.Hoffm. in Bot. Jahrb. Syst. 38(2): 211 (1906) [basionym].
**Ind. Loc.**: [Tanzania] Usambara: Gale; auf nassen Hochgebirgswiesen im Quellgebiet des Kwasindo, um 1200 m, im ganzen Wambugu-Lande häufig (BUCHWALD n. 318)—Blühend und fruchten im Dezember 1895). Nyassaland: Uhehe, Utschungwe-Berge, um 1600 m ü. M. (Frau Hauptmann PRINCE.—Blühend 1899).
**Lectotype (designated here):** [Tanzania] Usambara, 06.1900, *Buchwald 318* (BM000924815!).
**Notes:** O. Hoffmann’s original materials were mainly kept in B herbarium, but most of the collections of this herbarium were destroyed. We designate, as a lectotype, a specimen that, although it is currently kept in BM, was acquired from B, as the label indicates “ex Museo botanico Berolinensi”.
**Examined material: Congo:** Kivu, slope of Mt. Visoke facing Mt. Mikeno, alt. 3100, 02.18.1975, *W.G. d’Arcy 7948* (MNHN-P-P0010147). **Etiopia:** Bale Prov., Web river, just W. of Dinshu, about 157 km E. of Shashamane, along the road to Goba, alt. approx. 3000 m, 07.24.1970, *J.J.F.E.Wilde 6805* (BR0000016273061). **Malawi:** N. Prov., Nkhata Bay Dist. Vipya Plateau, 23 mi. SW of Mzuzu, Lwafa drift, alt. approx. 1675 m, 05.15.1976, *J.Pawek 11273* (WAG.1322534). **Rwanda:** Northern Province, Volcanoes National Park, Mount Gahinga, swampy area in a clearing of the bamboo forest, 02.08.2022, *M.Galbany-Casals, J.A.Calleja & M.Kandziora 2762*. **Tanzania:** Iringa, Makete District. Kitulo Plateau, 03.01.1991, *H.Suleiman, M.J.Fundi 13* (WAG.1322531); Iringa; Kitulo, Kitulo Plateau, 02.10.1969, *J.Prins-Lampert 166* (WAG.1534453/4).
***Afrocirsium schimperi*** **(Vatke) Calleja, Garcia-Jacas, Moreyra & Susanna, comb. nov.**
≡ *Cnicus schimperi* Vatke in Linnaea 39(5): 511 (1875) [basionym] ≡ *Cirsium schimperi* (Vatke) C.Jeffrey in Cufod., Bull. Jard. Bot. Natl. Belg. 37(3, Suppl.): 1174 (1967).
**Ind. Loc.:** [Ethiopia] in rivulorum ripa et in aqua ipsa ad Gaffat 8100′ a. m. Sept. 1863. (n. 1238). Eadem in coll. a. 1853. n. 36.
**Lectotype** (designated by Jeffrey, 1968): [Ethiopia] Abyssinia, au Bachufern im wasser 8100′ uber mur Gaffat, 25.09.1863, *Schimper 1238* (K000418422!). **Isolectotypes:** BM 000924814!, E 00611482! [photo], GRA0003185-0! [photo], S10-1686! [photo].
**Examined material: Ethiopia:** About 5 km NE of Addis Ababa, behind Italian Embassy, alt. approx. 2600 m, 10.16.1965, *W.J.J.O.de Wilde & de Wilde-Duyfjes B.E.E. 8280*, (BR0000016273092); Gojam: 4 km N of Debre Tabor along the old, now completely disused road to Addis Zemen, alt. 2550 m, 09.13.2004, *I.Friis, G.S.Bidgood, A.Hailu, B.Yitbarek 11553* (WAG.1534438); about 5 km NE. of Addis Abeba. Behind Italian Ambassy, alt. approx. 2600 m, *W.J.J.O.de Wilde & B.E.E.Wilde-Duyfjes 8280* (MNHN-P-P0010150); Debr’Eski, alt. 8000 m, 11.02.1851, *W.Schimper 36* (MNHN-P-P0010151).
***Afrocirsium straminispinum*** **(C.Jeffrey) Calleja, Garcia-Jacas, Moreyra & Susanna, comb. nov.**
≡ *Cirsium polyacanthum* Hochst. ex A.Rich., Tent. Fl. Abyss. 1: 456 (1848) [basionym], nom. illeg., non Kar. & Kit. (1841) ≡ *Cirsium straminispinum* C.Jeffrey in Cufod., Bull. Jard. Bot. Natl. Belg. 37(3, Suppl.): 1174 (1967).
**Ind. Loc.:** [Ethiopia] Crescit prope *Tchélatchékanné* non procul a convalle fluvii *Taccazé*, mense Julio (Quartin Dillon) et prope *Demerki*, in provincia *Semiène* (Schimper).
**Lectotype (designated here):** [Ethiopia] Abyssinie, Demerki, 09.08.1838, *Schimper 1147* (P0010166! [photo]). **Isolectotypes:** P0010167! [photo], P0010168! [photo], P0010169! [photo], BR0000008360465! [photo], LG0000090028557! [photo], K000418441!, S10-1669! [photo]).
**Examined material:** Ethiopia, Abyssinie, Tchélatchékanné, 1844, *MM.Quartin-Dillon et Petit s.n.* (P0010162! [photo], P0010163! [photo], P0010164! [photo], P0010165! [photo], P0010170! [photo]).
***Nuriaea* Susanna, Calleja & Moreyra, gen. nov.**
Spiny perennial herbs, (2.5) 3–4 (5) m high. Stems interruptedly spiny-winged, fistulose, sulcate, densely pilose in the grooves. Basal leaves very large, to 1 m long, cauline smaller to 40 cm, bipinnatisect, with basalmost lobes transformed in a stout spine’s 2 mm diam. and 3–4 cm length, sparsely pilose above, white–tomentose below. Capitula terminal in loose panicles, globose–umbilicate, 4–7 cm wide, homogamous. Involucral bracts glabrous; outermost bracts patent or reflexed, with a scariose triangular appendate, purple in *N. engleriana*, green in *N. dender*; middle bracts with a more developed appendage, entire in *N. engleriana*, pinnulate–spiny in *N. dender*. Florets white, or pink-purple, 5–6 cm long including the ovary; anther filaments papillose. Achenes lanceolate, compressed, glabrous, smooth or somewhat striate, 1 cm long, with a short apical coronule; apical caruncle absent. Pappus to 5 cm long; setae plumose. Type: *Nuriaea engleriana* (O. Hoffm.) Susanna, Calleja & Moreyra.
The name of the genus honors our coauthor Núria Garcia-Jacas, who passed away on April the 28th 2023, with this paper almost finished. Rest in peace.
***Nuriaea engleriana* (O. Hoffm.) Susanna, Calleja & Moreyra, comb. nov.**
≡ *Cirsium englerianum* O. Hoffm. in Bot. Jahrb. Syst. 38(2): 210 (1906) [basionym].
**Ind. Loc.:** Gallahochland: im Lande der Arussi-Galla, im Uferwald am Awala-See (Dr. ELLENBECK n. 1715.—Blühend im Dezember 1900).
**Neotype** (designated by Friis in Norweg. J. Bot. 22 [3]: 203 (1975)): Ethiopia, Kaffa Prov. S.E. of Folla, some 15 km N og Ghibe bridge on Addis-Jimma road, 7°52′ N, 37°11′ E, 2000–2100 m a.s.l., in shrub grassland on edge of path, 12.02.1970, Danish Botanical Expedition to Ethiopia 1970 (*I.Friis, A.Hounde & K.Jacobsen*) 551 (C100000339! [photo]). **Isoneotypes** (designated by Friis in Norweg. J. Bot. 22 [3]: 203 (1975)): BR0000005537143! (photo), ETH000000309! (photo), WAG 0258805! (photo).
**Notes:** O. Hoffmann’s original materials were originally kept in B but were destroyed. Due to the lack of duplicates, Friis (1975) designated a neotype and isoneotypes.
**Examined material: Ethiopia:** Bonga near R.C. Mission, alt. approx. 1800 m, *W.J.J.O.de Wilde & B.E.E.de Wilde-Duyfjes 9440*, 12.22.1965 (BR0000016273047, WAG.1322605); Kaffa prov. around Giren, alt. 2000 m, *I.Friis, A.Getachew, F.Rasmussen & K.B.Vollesen 1556*, 12.05.1972 (BR0000016273023).
***Nuriaea dender* (Friis) Susanna, Calleja & Moreyra, comb. nov.**
≡ *Cirsium dender* Friis in Norweg. J. Bot. 22(3): 203 (1975) [basionym].
**Holotype:** Ethiopia, Kaffa Prov., Mt. Maigudo, ca. 40 km from Jimma-Addis road on Omo-Nadda track, 7°30′ N 37°23′ E, 2650 m a.s.l., *Erica-Hagenia-Arundinara-Maesa-Agauria-Ilex*-scrub, along the road, 12.03.1972, Danish–Ethiopian Botanical Expedition 1972-73 (*I.Friis, G.Aweke, F.Rasmussen & K.Vollesen*) 1444 (C10000338! [photo]). **Isotypes:** BR0000008872876! and BR0000008872227! (photo), ETH000000080! (photo), K000418444!, WAG 000541! and WAG 0000542!.
**Examined material: Ethiopia:** Illubator Region, 55 km north of Tepi, along the new road to Gore, between Gecha and Macha, alt. 2200 m, *I.Friis, G S.Bidgood, P.Host, D.Desissa & S.Kebede 7161*, 11.15.1995 (BR0000016273009); Gamu-Gofa, in the mountains above Arba Minch near Chencha, alt. 2300 m, *I.Friis, G.S.Bidgood, M.Wondafrash & G.Gibre-Hiwot 8788*, 12.25.1997 (WAG.1322547); Kefa, Mt. Maigudo, ca. 40 km from Jimma-Addis road on Omo-Nadda track, alt. 2650 m, 12.03.1972, *I.Friis, G.Aweke, F.Rasmussen & K.B.Vollesen 1444* (WAG0000541/42).

**Key to Subtribe Carduinae**

***1***  Large plants more than 3 m high; globose capitula to 4–7 cm diam………………………***2***
***1***  Smaller plants, capitula usually less than 4 cm…………………………………………***4***
***2***  Stems to 5 m; leaves green above, white tomentose beneath; involucral bracts with flat apex; achenes linear–lanceloate; pappus plumose to 4 cm long…………………***Nuriaea***
***2***  Stems to 3 m; leaves uniformly glabrous or pilose; achenes obpyramidal or globose……………………………………………………………………………………………………………………***3***
***3***  Leaves white–variegated, conspicuously white–marbled along the veins above, with long–spiny, recurvate involucral bracts to 5 (7), the basalmost ones erect or reflexed; achenes oblong–ovoid; pappus barbellate……………………………………………..***Silybum***
***3***  Leaves not white–variegated or conspicuously white–marbled; spines of the invlucral bracts usually straight; achenes obpyramidal; pappus plumose………………..***Cynara***
***4***  Annual plants with pinnatisect leaves; lobes of the leaves linear, spiny; peripheral florets sterile and showy……………….……………………….…………………….***Galactites***
***4***  Annual or perennial plants without showy sterile peripheral florets………………….***5***
***5***  Pappus barbellate or scabrid; stamen filaments laterally acrescent…………………..***6***
***5***  Pappus plumose; stamen filaments free…………………………………………………***8***
***6***  Pappus bristles s-shaped; capitula less than 3 cm diam. on long leafless peduncles……………….……………………………….……………………….……………..***Tyrimnus***
***6***  Pappus bristles straight; peduncles foliose………………….…………………….…….***7***
***7***  Achenes with 10–15 longitudinal grooves; papillary hairs of the staminal filaments to 0.75 mm; epidermal cells on the dorsal corolla lobes undulate……………………***Carduus***
***7***  Achenes with four lines; papillary hairs of the staminal filaments to 0.13 mm; epidermal cells on the dorsal corolla lobes straight……………………………………***Afrocarduus***
***8***  Leaves white–veined; involucral bract apex with entire spine; corolla more deeply split on the abaxial side; apical elaiosome absent……………….………………….***Notobasis***
***8***  White–veined leaves absent; corolla regularly split; apical elaiosome present……..***9***
***9***  Involucral bracts pectinated on the margin and the appendix, rarely toothed…….***10***
***9***  Involucral bracts entire or dentate…………………….………………………………….***11***
***10***  Divaricately branched plants; achenes linear; capitula concealed by densely araneous hairs………………….….………………………………….…………….……………..***Picnomon***
***10***  Unbranched plants; capitula not concealed by bracts……………………….……………………….…………….***Afrocirsium***
***11***  Achenes broadly ovoid, not compressed, without nectary………………..***Ptilostemon***
***11***  Achenes oblong, compressed, with apical nectary……………………….………………***12***
***12***  Leaves green above, white–lanuginose beneath; elaiosome cylindrical…***Lamyropsis***
***12***  Leaves glabrous or uniformly pilose; elaiosome globose……………………….***Cirsium***

## Figures and Tables

**Table 1:** Summary of the most accepted previous generic delimitation in the *Carduus-Cirsium* group and the new classification proposed here.

Previous Classification	*n*° of Accepted Species	New Classification Proposed Here	*n*° of Species
*Carduus* L.	ca. 100	*Afrocarduus* (Kazmi) Garcia-Jacas, Moreyra & Susanna	10
		*Carduus* L.	ca. 90
*Cirsium* Mill.	ca. 450	*Afrocirsium* Calleja, Garcia-Jacas, Moreyra & Susanna	3
		*Cirsium* Mill.	ca. 450
		*Nuriaea* Susanna, Calleja & Moreyra (out of *Carduus-Cirsium* group)	2
*Notobasis* (Cass.) Cass.	1	*Notobasis* (Cass.) Cass.	1
*Picnomon* Adans.	1	*Picnomon* Adans.	1
*Silybum* Adans.	2	*Silybum* Adans.	2
*Tyrimnus* Cass.	1	*Tyrimnus* Cass.	1

## Data Availability

Raw sequencies generated during this study were deposited in the NCBI Short Read Archive database (SRA) under the BioProject accession number PRJNA957074 (access on 13 July 2023: https://www.ncbi.nlm.nih.gov/bioproject/?term=PRJNA957074). BioSample Accession numbers for each sample can be found in the Supplementary Materials along with herbaria codes and voucher information (Table S1).

## Supplementary Materials

The following supporting information can be downloaded at: https://www.mdpi.com/article/10.3390/plants12173083/s1, Figure S1: Pairwise distance histogram; Figure S2: Plastidic ML tree; Table S1: Vouchers and Accession numbers, Table S2: Morphological characters examined.
