# Supplementary material for: African Mountain Thistles: Three New Genera in the Carduus-Cirsium Group
Source: Plants (Basel). 2023 Aug 28;12(17):3083. doi: 10.3390/plants12173083 (PMC10489743; doi:10.3390/plants12173083)
Supplement: Supplementary file 1 [file plants-12-03083-s001.zip › Supplementary/Supplementary material.pdf]

Supplementary material

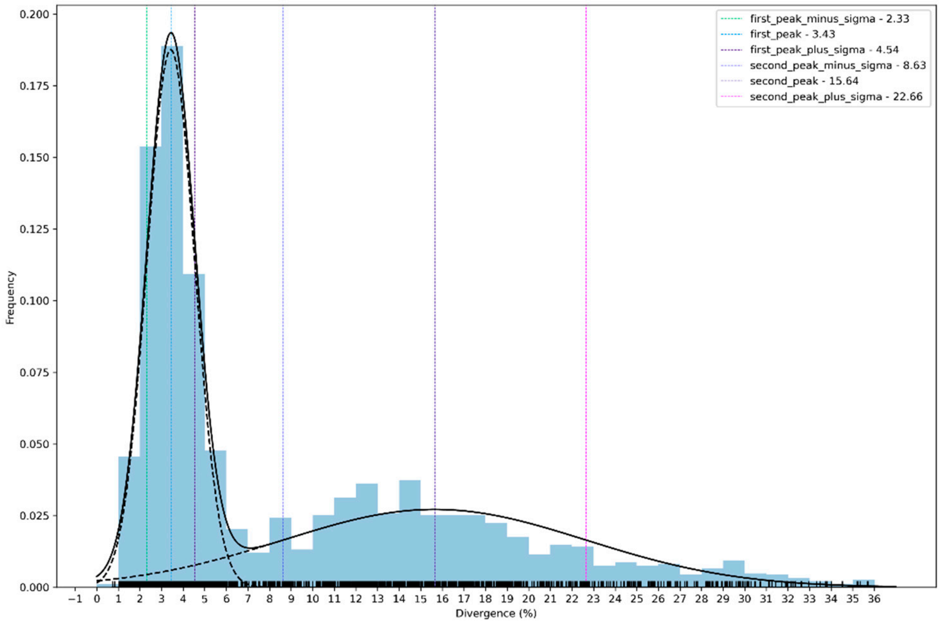

Figure S1. Pairwise distance histogram generated with ParalogWizard. The distribution of pairwise distances between exonic contigs for each locus is shown; distances are given as divergence percentage values (8.63-22.6%). The position of the first and second peak is indicated. The mean divergence and the standard deviation (plus and minus sigma) for each peak are marked by dashed lines; values are provided in the legend.

A

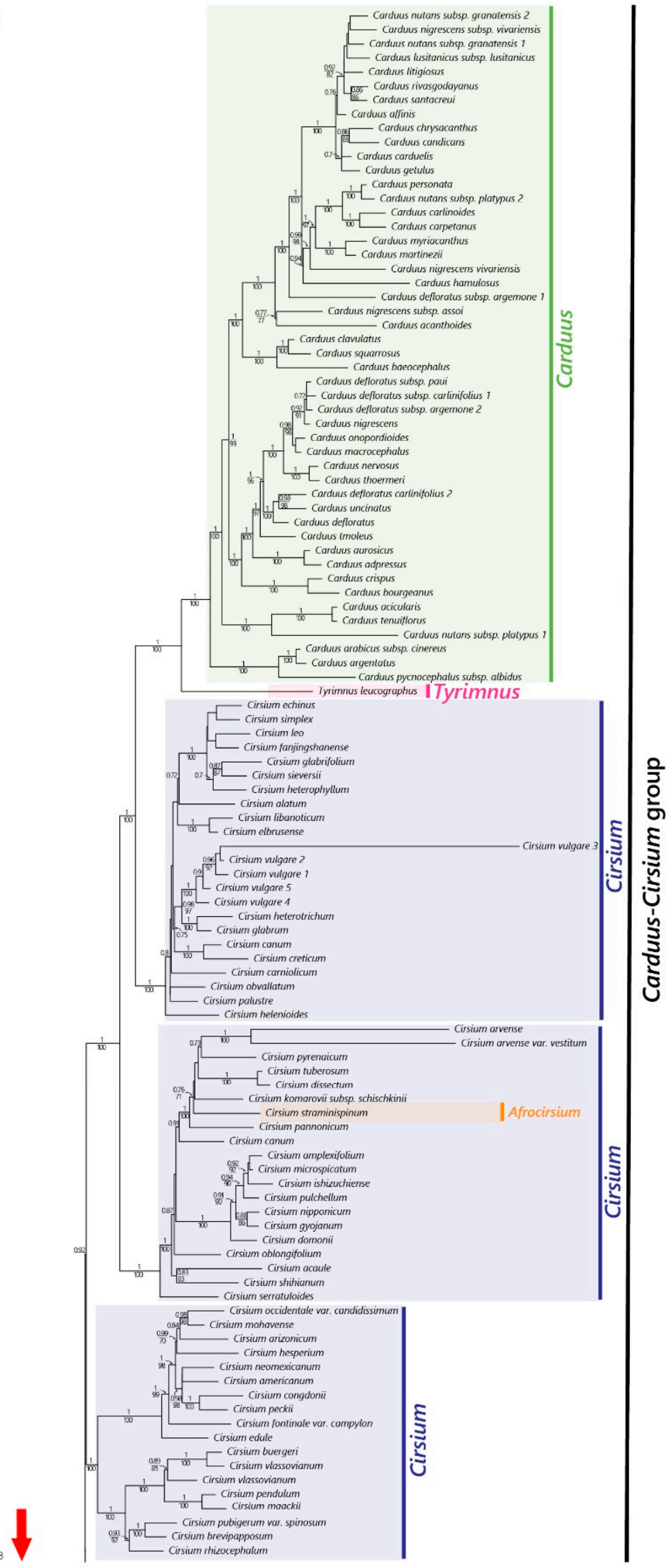
Carduus nutans subsp. granatensis 2
Carduus nigrescens subsp. vivariensis
Carduus nutans subsp. granatensis 1
Carduus lusitanicus subsp. lusitanicus
Carduus litigiosus
Carduus rivasgodayanus
Carduus santacreui
Carduus affinis
Carduus chrysacanthus
Carduus candicans
Carduus carduelis
Carduus getulus
Carduus personata
Carduus nutans subsp. platypus 2
Carduus carlinoides
Carduus carpetanus
Carduus myriacanthus
Carduus martinezii
Carduus nigrescens vivariensis
Carduus hamulosus
Carduus defloratus subsp. argemone 1
Carduus nigrescens subsp. assoi
Carduus acanthoides
Carduus clavulatus
Carduus squarrosus
Carduus baeocephalus
Carduus defloratus subsp. paui
Carduus defloratus subsp. carlinifolius 1
Carduus defloratus subsp. argemone 2
Carduus nigrescens
Carduus onopordioides
Carduus macrocephalus
Carduus nervosus
Carduus thoermeri
Carduus defloratus carlinifolius 2
Carduus uncinatus
Carduus defloratus
Carduus tmoleus
Carduus aurasicus
Carduus adpressus
Carduus crispus
Carduus bourgeanus
Carduus acicularis
Carduus tenuiflorus
Carduus nutans subsp. platypus 1
Carduus arabicus subsp. cinereus
Carduus argentatus
Carduus pycnocephalus subsp. albidus
Carduus
Tyrimnus leucographus
Tyrimnus
Cirsium echinus
Cirsium simplex
Cirsium leo
Cirsium fanjingshanense
Cirsium glabrifolium
Cirsium sieversii
Cirsium heterophyllum
Cirsium alatum
Cirsium libanoticum
Cirsium elbrusense
Cirsium vulgare 3
Cirsium vulgare 2
Cirsium vulgare 1
Cirsium vulgare 5
Cirsium vulgare 4
Cirsium heterotrichum
Cirsium glabrum
Cirsium canum
Cirsium creticum
Cirsium carniolicum
Cirsium obvallatum
Cirsium palustre
Cirsium helenioides
Cirsium
Carduus-Cirsium group
Cirsium arvense
Cirsium arvense var. vestitum
Cirsium pyrenaicum
Cirsium tuberosum
Cirsium dissectum
Cirsium komarovii subsp. schischkinii
Cirsium straminispinum
Afrocirsium
Cirsium pannonicum
Cirsium canum
Cirsium amplexifolium
Cirsium microspicatum
Cirsium ishizuchiense
Cirsium pulchellum
Cirsium nipponicum
Cirsium gyojanum
Cirsium domonii
Cirsium oblongifolium
Cirsium acaule
Cirsium shihianum
Cirsium serratuloides
Cirsium
Cirsium occidentale var. candidissimum
Cirsium mohavense
Cirsium arizonicum
Cirsium hesperium
Cirsium neomexicanum
Cirsium americanum
Cirsium congdonii
Cirsium peckii
Cirsium fontinale var. campylon
Cirsium edule
Cirsium buergeri
Cirsium vlassovianum
Cirsium vlassovianum
Cirsium pendulum
Cirsium maackii
Cirsium pubigerum var. spinosum
Cirsium brevipapposum
Cirsium rhizocephalum
Cirsium
B

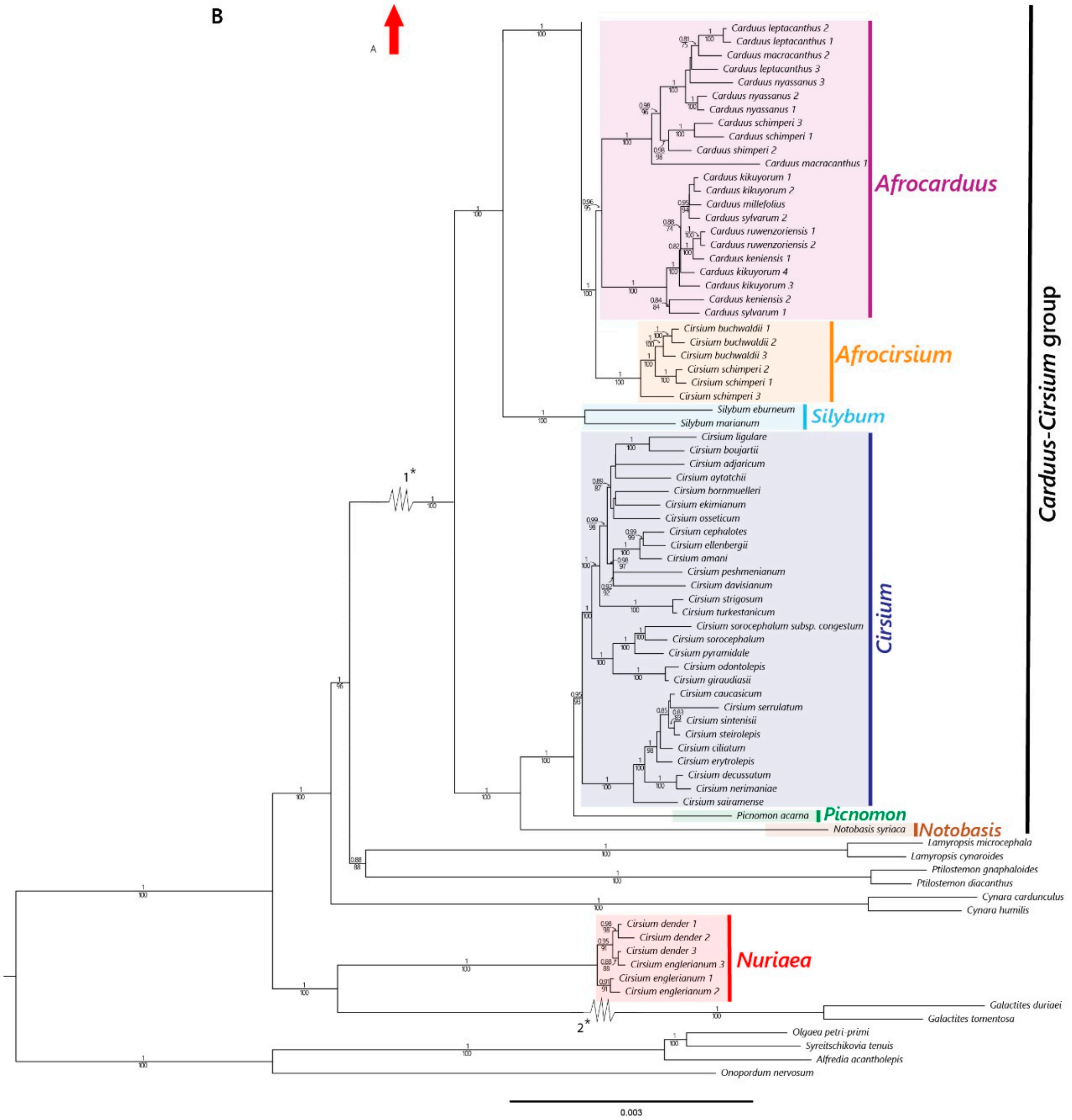

Figure S2. A) Maximum likelihood phylogenetic reconstruction for the subtribe Carduinae obtained with the chloroplast dataset. Values above branches indicate Transfer Expected Bootstrap (TBE) and those below branches indicate Felsenstein's bootstrap (BS); only values above 70% are shown. B) Maximum likelihood phylogenetic reconstruction for the subtribe Carduinae obtained with the chloroplast dataset. Values above branches indicate Transfer Expected Bootstrap (TBE) and those below branches indicate Felsenstein's bootstrap (BS); only values above 70% are shown. Branches shortened for fit are indicated on the phylogeny as 1* (50% of reduction) and 2* (30% of reduction).
